# A systematic review of cancer caregiver interventions: Appraising the potential for implementation of evidence into practice

**DOI:** 10.1002/pon.5018

**Published:** 2019-03-07

**Authors:** Anna Ugalde, Cadeyrn J. Gaskin, Nicole M. Rankin, Penelope Schofield, Anna Boltong, Sanchia Aranda, Suzanne Chambers, Meinir Krishnasamy, Patricia M. Livingston

**Affiliations:** ^1^ School of Nursing and Midwifery Deakin University Geelong Victoria Australia; ^2^ Faculty of Health Deakin University Geelong Victoria Australia; ^3^ Cancer Research Division Cancer Council NSW Sydney New South Wales Australia; ^4^ Department of Psychology Swinburne University Melbourne Victoria Australia; ^5^ Department of Cancer Experiences Research Peter MacCallum Cancer Centre Parkville Victoria Australia; ^6^ Sir Peter MacCallum Department of Oncology University of Melbourne Parkville Victoria Australia; ^7^ Strategy and Support Division Cancer Council Victoria Melbourne Victoria Australia; ^8^ Victorian Comprehensive Cancer Centre Parkville Victoria Australia; ^9^ Cancer Council Australia Sydney New South Wales Australia; ^10^ Faculty of Health University of Technology Sydney Sydney New South Wales Australia; ^11^ Cancer Council QLD Brisbane Queensland Australia; ^12^ Centre for Cancer Research University of Melbourne Parkville Victoria Australia

**Keywords:** cancer, caregiver, carer, dissemination, framework, implementation, intervention, oncology, outcomes

## Abstract

**Objective:**

nformal caregivers provide substantial support for people living with cancer. Previous systematic reviews report on the efficacy of cancer caregiver interventions but not their potential to be implemented. The aim of this systematic review was to explore the potential for cancer caregiver interventions to be implemented into practice.

**Methods:**

We searched three electronic databases to identify cancer caregiver interventions on 5 January 2018. We operationalised six implementation outcomes (acceptability, adoption, appropriateness, feasibility, fidelity, and costs) into a tool to guide data extraction.

**Results:**

The search yielded 33 papers (27 papers from electronic databases and six papers from other sources) reporting on 26 studies that met review criteria. Fewer than half the studies (46%) contained evidence about the acceptability of interventions from caregivers' perspectives; only two studies (8%) included interventions developed with input from caregivers. Two studies (8%) addressed potential adoption of interventions, and no studies discussed intentions, agreement, or action to implement interventions into practice. All studies reported on intervention appropriateness by providing a rationale for the interventions. For feasibility, on average less than one‐third of caregivers who were eligible to be involved consented to participate. On fidelity, whether interventions were conducted as intended was reported in 62% of studies. Cost data were reported in terms of intervention delivery, requiring a median time commitment of staff of 180 minutes to be delivered.

**Conclusions:**

Caregiver intervention studies lack components of study design and reporting that could bridge the gap between research and practice. There is enormous potential for improvements in cancer caregiver intervention study design to plan for future implementation.

## 1 BACKGROUND

Informal caregivers provide substantial practical and emotional support for people living with cancer, and in doing so, many receive minimal support themselves. Previous studies have outlined the negative impacts associated with being a caregiver, including depression,^1^ burden,^2^ social isolation,^3^ loss of self‐identity,^4^ sleep deprivation,^5^ financial burden,^6^ and significant changes to their lives.^2^ The role they take on in caring for the person with cancer is extensive, demanding, and often without training or resources.^7^

Many research papers focus on the development and evaluation of interventions aimed at improving the experience of caregivers, including several reviews of caregiver interventions.^8^, ^9^, ^10^, ^11^, ^12^, ^13^, ^14^, ^15^, ^16^ Of these, Northouse et al presented a meta‐analysis of the efficacy of caregiver intervention studies categorising interventions as psychoeducational, skills training, and therapeutic counselling. They concluded that interventions had beneficial small to medium effects on burden, coping, self‐efficacy, and quality of life.^15^ More recently, Ferrell and Wittenberg^11^ performed an updated review, identifying an increase in trials and the growing need to translate evidence into practice. Similarly, a review article drawing upon the literature and stakeholder perspectives from an in person meeting attended by more than 75 invited researchers, clinicians, advocates, and representatives recommended the implementation of successful interventions.^17^

Previous reviews have focused on the efficacy of caregiver interventions but not their potential to be implemented into practice. Implementation science frameworks contribute to reducing the evidence to practice gap^18^ by applying a theory to identify factors that may evaluate implementation success.^19^ Proctor et al^26^ developed a framework of implementation outcomes, defined as the “effects of deliberate and purposive actions to implement new treatments, practices and services” (p65). This framework has eight implementation outcomes: acceptability, adoption, appropriateness, (implementation) costs, feasibility, fidelity, penetration, and sustainability. Of these, the first six are relevant to the earlier stages of implementation, whereas penetration is relevant mid‐implementation and sustainability applies to longer‐term implementation. This framework has been applied to inform a variety of research topics including standardised multidisciplinary team meeting templates,^20^ shared decision‐making,^21^ cervical cancer prevention,^22^ and uptake of human papillomavirus (HPV) vaccines.^23^

Caregiver interventions show promise for potential implementation into practice.^11^, ^15^ However, we know little about whether interventions are designed and reported in a way that supports implementation.^24^, ^25^ There is a need to explore the implementation potential of existing cancer caregiver intervention studies to guide the development of future caregiver research. The aim of this review is to describe and appraise the cancer caregiving literature to explore the potential for caregiver interventions to be implemented into practice.

## 2 METHOD

### 2.1 Search strategy

This systematic review was registered on PROSPERO, number: CRD42018098838.

To identify studies for inclusion in this review, three electronic databases were searched, Cumulative Index to Nursing and Allied Health Literature (CINAHL) Complete, MEDLINE Complete, and PsycINFO Complete, representing the fields of nursing, medicine, and psychology. The terms used in the search were *caregivers* (as a subject heading) and *cancer*. No limitations were applied for language or publication date. The search was performed on 5 January 2018. The reference lists of papers meeting the inclusion criteria were scanned for additional papers for possible inclusion in the review.

We also searched reference lists of eight recent systematic reviews on caregivers of people with cancer.^8^, ^10^, ^11^, ^12^, ^13^, ^14^, ^15^, ^16^

### 2.2 Selection criteria

Studies were included in this review if they met the following criteria: (i) Participants were informal (unpaid) adult (18+ y) caregivers who had an active role in the provision of care for an adult with cancer; (ii) interventions were programmes, supports, sessions, or resources provided to, and directed towards supporting, caregivers to improve their own functioning or assist them in providing support for the patient (eg, programmes focusing on upskilling caregivers); (iii) study designs included at least two conditions (eg, randomised controlled trials and quasi‐experimental studies), one of which must have been a control condition (eg, active controls, waiting list controls, and treatment as usual [TAU] controls); and (iv) study outcomes focused on the caregiver. Pilot and feasibility studies were eligible for inclusion.

Studies were excluded if they met the following criteria: (i) They focused on spouses or other family without establishing that they had caregiving roles; (ii) 25% or more of patients had conditions other than cancer; (iii) the interventions focused on both patients and caregivers (interventions where minimal content was delivered to patients were eligible for inclusion, however); and (iv) the study design included two or more experimental conditions without a control condition. These exclusion criteria were established to ensure a focus on cancer caregiver interventions. Review papers were excluded from selection.

### 2.3 Study selection

Two authors (A.U. and C.J.G.) performed the eligibility assessment independently in an unbiased standardised manner. C.J.G. undertook an initial screening of papers, on the basis of title and then abstract. Both A.U. and C.J.G. then assessed papers on the basis of a full‐text review. Disagreements between reviewers were resolved through consensus. Deferring to a third reviewer was not necessary.

### 2.4 Data extraction

From each study meeting the selection criteria, data were extracted on study characteristics and the implementation outcomes of the interventions. Data extracted on study characteristics included (i) country of origin, (ii) aim, (iii) caregiver demographic characteristics (sample size, sex, and age), (iv) patient diagnosis, (v) relationship between caregiver and patient, (vi) study design, (vii) intervention details (format, content, setting, and who delivered the intervention), (viii) theory underpinning intervention (explicit statement required), (ix) evidence of prior pilot testing of intervention, (x) comparison condition, (xi) outcome measures, (xii) key findings, and (xiii) whether the conclusions were supported.

Operationalisation of the Proctor et al^26^ taxonomy of implementation outcomes (acceptability, adoption, appropriateness, feasibility, fidelity, and cost) guided the extraction of data on intervention implementation outcomes (see Table 1). This framework was selected because of the alignment between the implementation outcomes and the aims of the review. We selected this framework in preference to others in the implementation science discipline as it draws on a conceptual framework that addresses a range of outcomes. The outcomes are defined in a comprehensive manner that facilitates measurement for the purposes of a systematic review.

**Table 1:** Operationalisation of Proctor's framework for implementation outcomes

Implementation Outcome	Operationalisation in This Systematic Review	Response Options
Acceptability	Data collected on intervention acceptability from the perspectives of caregivers	Y/N/Partially/Possibly
	Data collected on intervention acceptability from the perspectives of other stakeholders	Y/N/Partially/Possibly
	Caregiver input into intervention development	Had input into intervention development/Caregivers informed the development/Not involved
Adoption	Evidence of intention, agreement, or action to try to employ intervention	Y/N; details
Appropriateness	Whether the intervention was a good fit	Y/N; details
	Whether the intervention was targeted to high risk caregivers	Y/N; details
Feasibility	Participation of caregivers screened:	Raw numbers, percentages, or not reported/not calculable
	• People screened	
	• Eligible	
	• Consented	
	• Commenced study	
	Participation of caregivers in the intervention condition:	Raw numbers, percentages, or not reported/not calculable
	• Withdrawal rate (choosing to no longer participate)	
	• Unable to complete intervention (ceasing involvement due to change in circumstances)	
	• Percentage who completed intervention (ie, they did not withdraw or were unable to complete)	
	Participant time commitment required for full intervention delivery	Time (minutes)
Fidelity	Whether the intervention ran as intended	Yes/No/Not reported; details
	Dose delivered	Percentage
	Changes to the intervention during the study	None reported/details
Costs	Staff time commitment required for delivery	Time in min/Not reported
	Additional resources used	None reported/details
	Staff training and expertise required to deliver intervention	Not specified/details

The framework was operationalised into a data collection tool by three authors (N.M.R., A.U., and C.J.G.). One author completed all data extraction (C.J.G.), with 20% of studies extracted by a second author (A.U.). Where necessary, two authors discussed ambiguities until consensus was achieved.

### 2.5 Data analysis

Descriptive statistics (frequencies, medians, and interquartile ranges [IQRs]) were used to summarise the data from the studies. Data were extracted to and analysed in Microsoft Excel.

## 3 RESULTS

**Figure 1:**
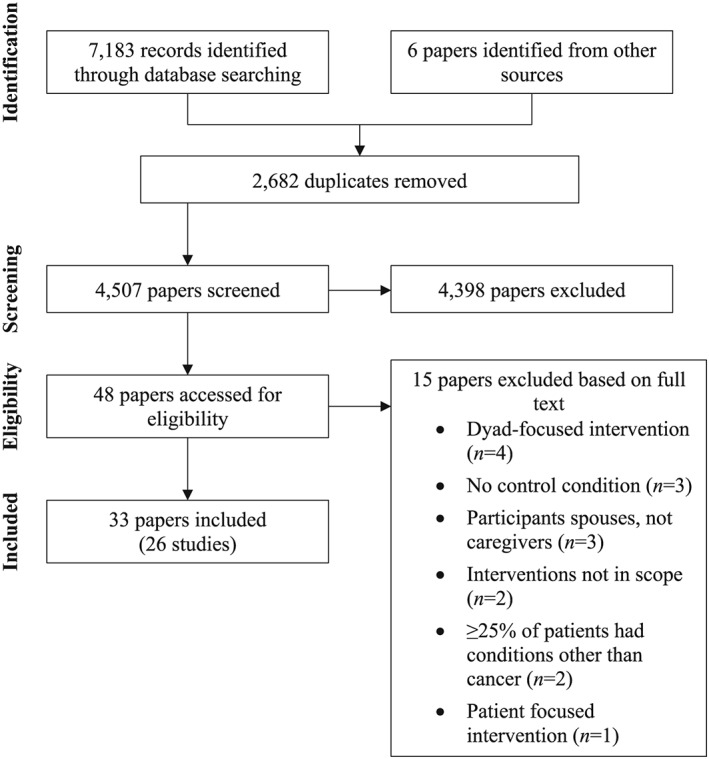
Identification and selection of studies for the systematic review

The search of electronic databases yielded 7183 records (CINAHL Complete, n = 2306; MEDLINE Complete, n = 2757; and PsycINFO Complete, n = 2120), of which 2682 were duplicates (see Figure 1). Of the remaining 4501 entries, 103 were retained following screening the titles of papers. After reviewing the abstracts, 61 papers did not meet the selection criteria and were excluded. The full texts of the remaining 42 papers were reviewed, of which 27 papers were finally included in the review.

An additional six papers meeting the selection criteria came from other sources. Inspection of the reference lists of previous systematic reviews in the area^8^, ^11^, ^12^, ^13^, ^14^, ^15^, ^16^ enabled identification of a further five papers that met the selection criteria. One further paper was identified from a preliminary search that abandoned because the search was too narrow. Being relevant, this paper was included in the review. Checking the reference lists of the included papers resulted in no further papers being identified for inclusion. In total, 33 papers were included in the review, representing 26 studies (Table 2).

**Table 2:** Study characteristics

Study	Country	Primary Aim	Participants	Patient Diagnosis	Caregiver‐Patient Relationship	Design	Comparison Condition	Main Outcome Measures	Key Results
Bahrami and Farzi (2014)^27^	Iran	To determine the effect of a supportive educational programme on the caring burden and quality of life in the family caregivers of women with breast cancer.	64 caregivers (64% female; exp: mean age = 37, SD = 11; con: mean age = 39, SD = 10)	Breast cancer	Husband (27%), child (48%), sibling (22%), parents (3%)	RCT	TAU	Caregiver burden, QOL	↓caregiver burden, ↑physical, mental, spiritual, and environmental domains and overall quality of life
Belgacem et al (2013)^28^	France	To assess the effect of a caregiver educational on patients' and caregivers' QOL and caregivers' burden.	67 caregivers (no descriptive statistics)	Haematological/other oncological cancer	Spouse (62%), offspring (17%), sibling (9%), parent (9%), friend (3%)	RCT	TAU	Caregiver burden, QOL	↑QOL (caregivers and patients), ↓burden (caregivers)
Bultz et al (2000)^30^	Canada	To trial of a brief psychoeducational group programme for partners of breast cancer patients.	34 partners (100% male; mean age = 51, range 32‐67)	Breast cancer (stage I or II)	Spouse/partner (100%)	RCT	Wait list	Mood, marital satisfaction, social support	↓mood disturbance (post‐intervention)
Carter (2006)^31^	USA	To test the feasibility and effectiveness of a brief behavioural sleep intervention for family caregivers of persons with advanced stage cancer.	30 caregivers (63% female; mean age = 53, SD = 17)	Advanced cancer	Spouse (57%), adult child (30%)	Repeated measures experimental design	Placebo	Sleep quality, depressive symptoms, QOL	↑sleep quality, ↓depressive symptoms
DuBenske et al (2014)^32^; Namkoong et al (2012)^51^	USA	To assess the impact of a web‐based lung cancer information, communication, and coaching system for caregivers versus the internet with a list of recommended lung cancer websites.	285 caregivers (68% female; mean age = 56, range = 18‐84)	Advanced NSCLC lung cancer	Spouse/partner (72%)	RCT	Active	Caregiver burden, disruptiveness, mood	↓burden, ↓negative mood; perceived bonding (i)positively affects caregivers' appraisal and problem‐focused coping strategies and (ii) mediates the effects of CHESS on coping strategies
Fegg et al (2013)^33^	Germany	To test the effectiveness of existential behavioural therapy on mental stress and quality of life.	133 caregivers (70% female; mean age = 55, SD = 13)	83% cancer	Partner (62%), parent (26%), child (3%)	RCT	TAU	Somatisation, anxiety, depression, satisfaction with life, quality of life	Post treatment: ↓anxiety, ↑QOL. 12 mo: ↓depression, ↑QOL
Hendrix et al (2013)^36^	USA	To investigate the effects of an individualised caregiver training programme on self‐efficacy in home care and symptom management.	120 caregivers (83% female; 85% aged 46+)	Haematological	Spouse (77%), child (13%)	RCT	Placebo	Caregiver self‐efficacy for managing patient symptoms and psychological well‐being (depression, anxiety, quality of life); patient physical symptoms	↑caregiver self‐efficacy
Hendrix et al (2016)^35^	USA	To compare the effects of the enhanced caregiver training (enhanced‐CT) protocol to an education condition with respect to caregiver self‐efficacy in cancer symptom management and stress management and preparedness.	138 caregivers (83% female; mean age = 55, SD = 13)	Cancer	Spouse (67%), child (15%), parent (10%)	RCT	Placebo	Caregiver self‐efficacy for managing patient symptoms, caregiver stress, preparedness for caregiving	↑caregiver self‐efficacy, ↓caregiver stress, ↑preparedness for caregiving (at post‐training, but not at 2‐ and 4‐wk post‐discharge)
Holm et al (2016)^37^; Holm et al (2017)^38^	Sweden	To evaluate the short‐term and long‐term effects of a psychoeducational group intervention designed to increase preparedness for family caregiving in specialised palliative home care.	194 caregivers (66% female; exp: mean age = 63, SD = 13; con: mean age = 60, SD = 14)	90% cancer	Spouse (48%), adult child (35%)	RCT	TAU	Preparedness for caregiving, competence for caregiving, rewards for caregiving, caregiver burden, health, anxiety, and depression	↑preparedness for caregiving upon completion of intervention and at 2‐mo follow‐up, ↑competence for caregiving upon completion of intervention; caregivers who did not benefit: ↑preparedness and competence for caregiving and their health at baseline, ↓environmental burden at baseline
Hudson et al (2005)^41^	Australia	To examine the effectiveness of a psychoeducational intervention to enhance the support and guidance offered to primary family caregivers of patients receiving home‐based palliative care for advanced cancer.	106 caregivers (65% female; mean age = 61, SD = 14)	Advanced cancer	Spouse (67%), child (16%), parent (8%)	RCT	TAU	Caregiver preparedness, mastery, self‐efficacy, competence, rewards, anxiety	↑caregiver rewards
Hudson et al (2013)^39^; Hudson et al (2015)^40^	Australia	To test the effects of a psychoeducational intervention on caregiver distress, unmet needs, preparedness, competence, and emotions.	298 caregivers (71% female; mean age = 59, SD = 14)	Advanced cancer	Not reported	RCT	TAU	Caregiver psychological distress, unmet needs, preparedness, competence, positive emotions	↑caregiver preparedness and ↑competence (for those receiving two home visits) between baseline and 1‐wk post‐intervention, and ↓caregiver distress (for those receiving one home visit) between 1‐wk post‐intervention and 8‐wk post‐death
Kurtz et al (2005)^42^; Given et al (2006)^34^	USA	To investigate whether a clinical nursing intervention focusing on teaching family caregivers and their cancer patients skills to better manage the patients' symptoms would reduce caregiver depressive symptomatology.	237 caregivers (54% female; mean age = 55, SD = 14)	Cancer	Not reported	RCT	TAU	Depressive symptoms, reactions to assisting with chemotherapy symptoms, involvement in symptom management	↓negative reactions to assisting with symptoms
Laudenslager et al (2015)^43^; Simoneau et al (2017)^55^	USA	To test the effects of a psychosocial intervention (psychoeducation, paced respiration, and relaxation [PEPRR]) on the distress of caregivers of allogeneic haematopoietic stem cell transplant (allo‐HSCT) patients.	148 caregivers (76% female; mean age = 54, CI = 52‐56)	Cancer, receiving allo‐HSCT	Spouse/partner (70%), parent (18%)	RCT	TAU	Caregiver distress, salivary cortisol awakening response	↓caregiver distress
Lee et al (2016)^44^	Taiwan	To test the ability of an integrative intervention programme for caregivers of advanced cancer patients to lower caregiving burden as death approaches.	81 caregivers (76% female; exp: mean age = 51, SD = 16; con: mean age = 50, SD = 14)	Advanced cancer	Spouse (58%), child (27%)	Repeated measures, two‐group comparative design	TAU	Subjective and objective burden	↓subjective burden, ↑caregiver self‐efficacy and objective burden
Leow et al (2015)^45^	Singapore	To evaluate the effectiveness of a psychoeducational intervention, the caring for the caregiver programme.	80 caregivers (68%; mean age = 47, SD = 12)	Advanced cancer (stage IV)	Child (58%), spouse (25%), sibling (4%), parent (3%), niece (1%), daughter‐in‐law (9%), grandchild (1%)	RCT	TAU	QOL, social support, stress, depression, self‐efficacy in self‐care, closeness with the patient, rewards of caregiving, knowledge	↑QOL, ↑social support, ↓stress, ↓depression, ↑self‐efficacy in self‐care, ↑closeness with the patient, ↑rewards of caregiving, ↑knowledge
Manne et al (2004)^47^	USA	To investigate the effects of a psychoeducational group intervention on the distress, coping, personal growth, and marital communication of wives of men diagnosed with prostate cancer.	60 caregivers (100% female; mean age = 60, SD = 9)	Prostate cancer	Spouse/partner (100%)	RCT	TAU	Caregiver distress (general and cancer‐specific), coping, post‐traumatic growth, cancer‐specific marital interactions	↑post‐traumatic growth
Mahendran et al (2017)^46^	Singapore	To evaluate the pilot COPE (Caregivers of cancer Outpatients' Psycho‐Education support group therapy) intervention.	97 caregivers (65% female; 52% aged 41‐60)	Cancer	Spouse (35%), child (33%)	Quasi‐experimental design without randomisation	Wait list	Depressive symptoms, anxiety, QOL	No significant effects
McMillan et al (2006)^49^; McMillan et al (2007)^48^	USA	To determine whether hospice plus a coping skill training intervention improved family caregivers' quality of life, burden, coping, and mastery, compared with hospice plus emotional support, and usual hospice care.	329 caregivers (85% female; exp: mean age = 63, SD = 14; con[TAU]: mean age = 60, SD = 15; con[support]: mean age = 62, SD = 15)	Advanced cancer	Not reported	RCT	Two comparison groups: TAU and placebo	Caregiver quality of life, caregiver burden due to patient symptoms, caregiver burden due to tasks, caregiver mastery	↑caregiver quality of life, ↓caregiver burden due to patient symptoms, ↓caregiver burden due to tasks
Mitchell et al (2013)^50^	Australia	To assess the efficacy of a general practitioner (GP)–based intervention incorporating a carer‐reported needs checklist and a supporting GP toolkit of resources, reduces the reported number and intensity of unmet carer needs.	392 caregivers (67% female; exp: mean age = 57, SD = 13; con: mean age = 58, SD = 13)	Advanced cancer (locally invasive or metastatic disease)	Spouse/partner (68%), parent (9%), adult child (15%), sibling (2%)	RCT	TAU	Unmet needs, anxiety, depression, quality of life	↑mental well‐being (at 6 mo, for those with baseline clinical anxiety), ↓anxiety (at 6 mo, for those with baseline clinical depression), ↑physical well‐being (at 1 mo, for those not anxious)
Pailler et al (2015)^52^	USA	To assess the feasibility, acceptability, and efficacy of a supportive group‐based intervention for family caregivers.	69 caregivers (57% female; mean age = 55, SD = 14)	Leukaemia	Spouse (64%), significant other (10%), parent (13%), other family (13%)	Pre‐post sequential cohort design	TAU	Distress (caregiver and patient), caregiver quality of life, satisfaction (with intervention)	↓caregiver distress, ↑caregiver quality of life
Rexilius et al (2002)^53^	USA	To examine the effect of massage therapy and healing touch on anxiety, depression, subjective caregiver burden, and fatigue experienced by caregivers of patients undergoing autologous haematopoietic stem cell transplant.	36 caregivers (72% female; mean age = 52 y)	People undergoing autologous haematopoietic stem cell transplant	Spouse (69%), sister (17%), mother (11%), fiancé (3%)	Pre‐post design with randomisation of groups (not individuals)	Placebo	Anxiety, depression, subjective burden, fatigue	Massage therapy: ↓anxiety, ↓depression, ↓fatigue; healing touch: no effects
Shaw et al (2016)^54^	Australia	To investigate the effectiveness of a structured telephone intervention for caregivers of people diagnosed with poor prognosis gastrointestinal cancer on psychosocial outcomes for both caregivers and patients.	128 caregivers (39% female; exp: mean age = 60, SD = 14; con: mean age = 64, SD = 14)	Gastrointestinal cancer	Spouse/partner (70%), child (23%), parent (2%), sibling (2%), other family member (1%), friend (2%)	RCT	TAU	Caregivers' quality of life (QOL), caregiver burden, unmet supportive care needs, and distress. Patients' QOL, unmet supportive care needs, distress, and health service utilisation	3 mo post hospital discharge: caregivers, ↑social support, ↓worry about finances; patients, ↓emergency department presentations, ↓unplanned hospital readmissions
Sun et al (2015)^56^	USA	To test the effectiveness of an interdisciplinary palliative care intervention for family caregivers of patients diagnosed with stage I through IV non–small cell lung cancer.	366 caregivers (62% female; exp: mean age = 58, SD = 14; con: mean age = 57, SD = 13)	Non–small cell lung cancer	Not reported	Prospective, sequential, quasi‐experimental design	TAU	Caregiver burden, caregiving skills preparedness, psychological distress, quality of life	↓caregiver burden, ↓psychological distress, ↑social well‐being
Toseland et al (1995)^57^; Blanchard et al (1996)^29^	USA	To assess the impact of a short‐term individual counselling programme for cancer caregivers.	78 caregivers (50% male; exp: mean age = 56; con: mean age = 51)	Cancer	Spouse (100%)	RCT	TAU	Perceived health status, psychological well‐being (depressed mood, anxiety, burden, stress), social support, coping behaviour	2 wk: no effects; 6 mo: ↓depressed mood
Tsianakas et al (2015)^58^	England	To test the feasibility and acceptability of a complex intervention for carers of patients starting chemotherapy.	43 caregivers (65% female; mean age = 53, range = 24‐76)	Breast, lung, or colorectal cancer	Spouse/partner (44%), child (26%), parent (5%), friend (7%), other relative (18%)	RCT and focus groups	TAU	Knowledge of chemotherapy and its side effects, experience of care, satisfaction with outpatient services, coping, emotional well‐being	↑understanding of symptoms and side effects, ↑frequency of information needs being met
Walsh et al (2007)^59^	England	To evaluate the effectiveness of increased support for distressed, informal carers of patients receiving palliative care.	271 caregivers (79%; mean age = 56, SD = 14)	Cancer	Spouse/partner (64%), child (25%), other (12%)	RCT	TAU	Caregiver distress, strain, quality of life, satisfaction with care, bereavement outcome	No significant effects

Abbreviations: RCT, randomised controlled trial; TAU, treatment as usual; QOL, quality of life.

### 3.1 Study characteristics

An overview of study characteristics is presented in Table 2. Intervention characteristics and implementation outcomes tables are attached as supporting information (Table S1).

#### 3.1.1 Country of origin

Almost half of the studies (n = 12, 46%) were conducted in the United States.^29^, ^31^, ^32^, ^34^, ^35^, ^36^, ^42^, ^43^, ^47^, ^48^, ^49^, ^51^, ^52^, ^53^, ^55^, ^56^, ^57^ Australia was the second largest contributor of studies (n = 4, 15%).^39^, ^40^, ^41^, ^50^, ^54^

#### 3.1.2 Participant characteristics

The median number of participants included in the studies was 113 (IQR = 68 to 226). The majority of participants were female in 22 of 26 studies (median = 67%, IQR = 63% to 76%). On average, two‐thirds of the caregivers were the patients' spouses/partners (median = 66%, IQR = 57% to 70%). In most studies (24 of 26), all patients had been diagnosed with cancer; of the remaining two studies^33^, ^37^, ^38^ 83% and 90% of patients had cancer (the remaining patients had other chronic conditions).

#### 3.1.3 Study design

Three quarters of studies (n = 20, 77%) were randomised controlled trials. The comparison condition in three quarters of studies (n = 19, 73%) was TAU, with placebo controls used in a further 19% (n = 5) of studies.^31^, ^35^, ^36^, ^48^, ^49^, ^53^

#### 3.1.4 Intervention design

Two‐thirds of interventions were delivered face‐to‐face to individual caregivers (n = 18, 69%), with 27% (n = 7) delivered face‐to‐face to groups^30^, ^33^, ^37^, ^38^, ^46^, ^47^, ^52^, ^58^ and 4% (n = 1) requiring caregivers to access the intervention independently through the internet.^32^, ^51^ In addition, a quarter of interventions incorporated supplementary material, such as handouts and DVDs (n = 6, 23%).^35^, ^36^, ^39^, ^41^, ^43^, ^52^, ^55^, ^58^

Half the interventions included information provision (n = 14, 54%). Content also included skills development (n = 8, 31%),^27^, ^28^, ^35^, ^36^, ^41^, ^43^, ^44^, ^45^, ^46^, ^48^, ^49^, ^55^ social support (n = 6, 23%),^32^, ^37^, ^38^, ^41^, ^45^, ^51^, ^52^, ^59^ individual and group therapy (n = 5, 19%),^29^, ^30^, ^31^, ^33^, ^46^, ^57^ and self‐care (n = 4, 15%).^31^, ^41^, ^43^, ^45^, ^53^, ^55^ These percentages exceed 100% because of many interventions having multiple types of content.

The settings of two‐thirds of the interventions were health services (n = 18, 69%). Interventions also took place via telephone (n = 8, 31%),^27^, ^34^, ^39^, ^40^, ^41^, ^42^, ^44^, ^45^, ^54^, ^59^ in caregivers' homes (n = 5, 19%),^32^, ^39^, ^40^, ^41^, ^45^, ^48^, ^49^, ^51^ and at places convenient for caregivers (n = 2, 8%).^31^, ^59^ These percentages exceed 100% because of some interventions being delivered in multiple settings.

Staff most commonly delivering the interventions were nurses (n = 13, 50%), social workers (n = 5, 19%),^37^, ^38^, ^43^, ^47^, ^55^, ^56^, ^57^ and psychologists (n = 4, 15%).^30^, ^46^, ^47^, ^54^

Theoretical frameworks underpinned the interventions in under half of the studies (n = 12, 46%). Bandura's conceptualisation of self‐efficacy was the most commonly used theory (n = 5, 19%).^34^, ^35^, ^36^, ^42^, ^44^, ^45^

Interventions had been previously piloted in a third of studies (n = 8, 31%).^33^, ^36^, ^39^, ^40^, ^41^, ^43^, ^44^, ^45^, ^46^, ^55^, ^59^ In two studies (8%), aspects of the intervention had been piloted.^35^, ^50^ In a further two studies (8%), the investigations were pilot studies.^45^, ^46^ For the remaining studies, we found that pilots had not been conducted (n = 4, 15%) or were not reported (n = 10, 38%).

### 3.2 Implementation outcomes

The implementation outcomes across studies are presented in Table S2 (acceptability, adoption, and appropriateness), Table S3 (feasibility), and Table S4 (fidelity and costs). Findings are summarised below.

#### 3.2.1 Acceptability

In almost half the studies (n = 12, 46%), there was no evidence to indicate the acceptability of interventions from caregivers' perspectives. For 11 studies (42%), acceptability data were reported, which were collected via surveys (n = 5),^30^, ^36^, ^52^, ^53^, ^59^ focus groups (n = 2),^37^, ^38^, ^58^ interviews (n = 2),^46^, ^54^ and engagement with the intervention or debriefing (n = 2).^31^, ^43^, ^55^ In one further study, acceptability data were collected on certain aspects of an intervention (ie, feedback was gathered on some aspects of the intervention but not others).^35^ For the two remaining studies, acceptability data may have been collected, but insufficient information was provided to enable firm judgements to be made.^45^, ^50^

Most studies (n = 21, 81%) did not report on the acceptability of interventions from the perspectives of other stakeholders (ie, stakeholders other than caregivers). These data were available for three studies and were collected via focus groups (n = 2)^37^, ^38^, ^58^ and surveys (n = 1).^30^ Stakeholder acceptability data were collected on some components of an intervention in one further study^50^ and may have been obtained in another study.^45^

Caregivers appeared to have limited input into intervention development. In most studies (n = 17, 65%), there was no evidence of caregiver involvement in the development of interventions. Caregivers were directly involved in the development of the intervention in one study (4%),^58^ and in eight studies (31%), caregivers were involved in separate studies, such as focus groups, that informed the interventions.^39^, ^40^, ^41^, ^43^, ^45^, ^46^, ^50^, ^54^, ^55^, ^59^

#### 3.2.2 Adoption

No studies reported on intentions, agreement, or action to implement interventions into practice. However, two studies reported issues about the potential adoption of the interventions. In one, researchers reported that health care providers held reservations about possible implementation,^28^ and in the other paper, researchers noted that the intervention may not be sustainable in the longer term because of the costs involved in delivery.^46^

#### 3.2.3 Appropriateness

In all studies, interventions were considered a good fit, with solid arguments presented as to why the interventions were appropriate for the cancer caregivers.

Few interventions were targeted towards specific population groups who may have high support needs or may benefit from interventions, such as caregivers experiencing high levels of distress or those from minority cultural backgrounds (n = 2, 8%).^31^, ^59^ One intervention targeted caregivers with high distress levels,^59^ and another one focused on caregivers who had experienced sleep difficulties for at least 1 month.^31^

#### 3.2.4 Feasibility

Most caregivers screened were eligible for inclusion in the studies (median = 84%, IQR = 52% to 90%, data available from 65% of studies). On average, less than one‐third of eligible caregivers consented to participate (median = 28%, IQR = 17% to 55%, from 69% of studies). Most caregivers who consented to be involved commenced the interventions.

Most caregivers allocated to intervention conditions completed the interventions (median = 92%, IQR = 86% to 100%, from 65% of studies). On average, few caregivers withdrew (median = 6%, IQR = 0% to 13%, from 65% of studies). In only four studies were some caregivers unable to complete the interventions (because of circumstances such as the death of a patient) (non‐completion ranged from 3% to 23% across these studies).^29^, ^32^, ^43^, ^54^, ^55^, ^57^

The time commitment necessary for caregivers to complete interventions ranged from 79 minutes^58^ to 22 hours^33^ (median = 180 min, IQR = 120 to 360 min, from 65% of studies). Six studies had interventions that took 6 hours or more to deliver.^29^, ^30^, ^33^, ^37^, ^38^, ^43^, ^47^, ^55^, ^57^

#### 3.2.5 Fidelity

In the majority of studies, interventions appeared to be conducted as intended (n = 19, 62%). In one study, researchers reported that caregivers did not engage with one aspect of the intervention (an online forum).^45^ For the remaining studies, no information about intervention fidelity was reported (n = 9, 35%).

On average, caregivers completed all aspects of the interventions, such as attending all sessions provided (median = 100%, IQR = 84% to 100%, from 54% of studies).

No changes to interventions during the studies were reported, and no changes to the dose, delivery, or strategies during the studies were reported.

#### 3.2.6 Costs

The time commitment data were available for n = 19 (62%) of studies. Time required of staff ranged from 79 minutes^58^ to 22 hours^33^ (median = 180 min, IQR = 120 to 360 min).

The additional resources used in the interventions included written material (n = 7, 27%),^35^, ^36^, ^41^, ^43^, ^48^, ^49^, ^50^, ^52^, ^55^, ^56^, ^58^ audio material (n = 2, 8%),^41^, ^52^ DVDs (n = 2, 8%),^45^, ^58^ laptop computers with internet access for participants who required them (n = 1, 4%),^32^, ^51^ biofeedback devices (n = 1, 4%),^43^, ^55^ and home help aides (n = 1, 4%).^48^, ^49^ In over half of the studies (n = 15, 58%), no additional resources were reported.

For most studies (n = 22, 85%), aside from the occupations (and, in some cases, experience) of those who delivered the interventions, no information was provided on the training and expertise required to deliver the interventions. In two studies (8%), staff training was provided,^33^, ^58^ and in a further two studies (8%), the training and experience required was unspecified.^27^, ^52^

## 4 DISCUSSION

With recent calls for a need to focus on implementation of interventions,^17^, ^60^, ^61^ this review aimed to explore the implementation potential of cancer caregiver intervention studies. Although the reviewed studies focused on efficacy, there is a need to design, conduct, and report research that can be implemented into practice.^24^ The main finding from this review was that studies were not designed or reported in a way to maximise the potential for interventions to be successfully implemented. We also gained insights about the challenges of operationalising implementation outcomes from an established framework.

Results varied across the six implementation outcomes. These studies had limited evidence of acceptability, with few studies reporting on whether interventions were considered appropriate or involved consumers in the design of the interventions. There was little evidence for adoption. There was mixed support for interventions being appropriate: Although all interventions were reported to be a good fit through alignment with caregiver need and previous research, very few studies targeted groups specifically in need of support. There was limited support for feasibility, with data not reported for many studies, and low enrolment of caregivers in interventions. There was evidence for good fidelity of interventions. Costs were mostly reported in terms of staff time to deliver interventions and in some cases specified an investment required for staff time, training, or resources.

This review suggests that the reporting of cancer caregiver intervention studies requires improvement to support implementation into practice. There appears to be two key issues. Firstly, studies were not designed in ways that would maximise their potential to be successfully implemented. Secondly, in other instances, there is limited information reported relevant to implementation. Restrictions in reporting research in terms of journal requirements and required word counts may limit the opportunity to report evaluation data that includes outcomes of relevance to implementation.

There are other key findings from this review to highlight. The first is that consumer input into intervention development was notably low (acceptability outcome). In performing this review, we differentiated between studies that had active engagement with consumers as part of the project design, those studies that had developed interventions that were informed by the research team identifying a need, and those that had no consumer involvement. Consumer involvement into interventions is considered best practice,^62^ and it was surprising to find a paucity of studies utilising caregiver input. Future research should engage caregivers as team members and promote active roles in the development and refinement of caregiver interventions.

A further finding was the tendency for studies to recruit broadly rather than targeting groups more in need of support. This is in the context of consent rates that, while varied, had a median of less than a third of those approached across the studies, meaning that while many caregivers were eligible, this did not translate to enrolment. A recent systematic review and meta‐analysis exploring the efficacy of psychological interventions on anxiety in cancer patients found that low psychological distress at baseline was a key reason for low effectiveness, with authors advocating for screening and assessment of anxiety as an inclusion criterion before enrolment in psychological interventions.^63^ Caregivers not experiencing a problem may have low motivation to spend time in an intervention study they see as not relevant to their situation. Others have noted the need to increased research for vulnerable caregiving populations and risk stratification to target those most in need of support.^17^ Targeting groups in need of support is an important avenue for future caregiver intervention research.

This review also found that while most caregivers screened were eligible (feasibility outcome), this frequently was not well reported. Future studies should clearly report about the participants who were assessed for eligibility in accordance with CONSORT criteria and flow charts.^64^ There was also limited evidence available about intentions, agreements, or actions to implement interventions into practice (adoption outcome). There could be various reasons for this including that adoption is regarded as being outside the scope of conduct and reporting of studies, with adoption frequently reported at 6, 12, or 18 months after initial implementation. The lack of funding to test implementation processes has been acknowledged.^65^ Information about adoption agreements with service providers or potential would be a useful addition to papers reporting trials of interventions, even when the focus is on efficacy.

This review has operationalised Proctor's implementation outcomes framework. While there are other potential frameworks,^19^, ^66^ this framework was selected as the six domains resonated with the scope of the review. In practice, the operationalisation and data extraction allowed for key information to be assessed and findings support this framework as being appropriate for this review. Frameworks can be used to plan and design studies to strengthen the potential for implementation,^24^ and there may be potential to build on these results and use the Proctor framework in this context. This could strengthen the implementation potential of new studies. A recent literature review has outlined instruments to assess implementation outcomes, and addition of these measures could be considered in future trials.^67^ We did not include two implementation outcomes: penetration (the integration of an intervention within its setting) and sustainability (extent to which an intervention is maintained) given these are longer‐term outcomes.^26^ This review focused on cancer caregiver interventions, and issues of implementation potential may not be unique to this content.

### 4.1 Study limitations

This review has limitations to consider. Firstly, this review focused on implementation potential utilising a specific framework applied to the reporting of the original trial, but this may not mean that interventions were not implemented into practice. Studies may show limited implementation potential according this extracted data but may have been successfully implemented into practice. It appears that there are few published reports around implementation of cancer caregiver interventions; however, it was beyond the scope of our review to ascertain this. A further limitation is that it is important to acknowledge the diversity of cancer caregiving interventions in the literature. We screened abstracts broadly, and criteria focused on specific cancer caregiver interventions; for example, we omitted interventions directed at caregiver and patient dyads. This criterion was applied to ensure these interventions were specifically for caregivers. This review was conducted in the context of numerous caregiving reviews focusing on efficacy, and our aim was to complement these through exploring implementation potential.

### 4.2 Clinical and research implications

There are numerous implications for future research. Exploring any relationship between implementation outcomes and efficacy of interventions was outside the scope of this review, but this could be relevant for future research to inform optimal delivery on implementation outcomes. Exploring the potential of the implementation outcomes framework to plan and design studies may lead to stronger potential for implementation for cancer interventions. Additionally, given the findings of this review, the development and conduct of high‐quality cancer caregiver interventions that are able to be implemented into practice is essential.

## 5 CONCLUSIONS

Interventions must be cost‐effective and accessible; planning for implementation is important.^24^ Our findings suggest that the reporting of cancer caregiver interventions demonstrates limited capacity to be translated into practice. This is of significant concern as it may indicate limited public health or clinical benefit. This review has outlined the need for future caregiver studies to include caregivers in the design of interventions and focus resources and time commitments to those who need support. The demonstrated evidence for efficacy of caregiver interventions has limited relevance if interventions are not designed or conducted in a way to support implementation into practice. This review identifies the challenges involved in closing the evidence‐practice gap and contributes to the growing body of knowledge on which actions are required to ensure successful interventions actually reach targeted population groups.

## CONFLICT OF INTEREST

The authors have no conflict of interest to declare.

## Supporting information

Supporting Table 1: Overview of interventions.
Supporting Table 2: The acceptability, adoption, and appropriateness of interventions.
Supporting Table 3: The feasibility of interventions.
Supporting Table 4: The fidelity and costs of interventions.
