# Supplementary material for: A systematic review of cancer caregiver interventions: Appraising the potential for implementation of evidence into practice
Source: Psychooncology. 2019 Mar 7;28(4):687–701. doi: 10.1002/pon.5018 (PMC6594143; doi:10.1002/pon.5018)
Supplement: Supplementary file 1 — Supporting Table 1: Overview of interventions. Supporting Table 2: The acceptability, adoption, and appropriateness of interventions. Supporting Table 3: The feasibility of interventions. Supporting Table 4: The fidelity and costs of interventions. [file PON-28-687-s001.docx]

Supporting Information files

Supporting Table 1: Overview of interventions.

| Study | Format | Content | Setting | Staffing | Theory Underpinning Intervention | Pilot Testing |
| --- | --- | --- | --- | --- | --- | --- |
| Bahrami & Farzi, 2014 | 4 individual sessions (2 hospital visits and 2 telephone sessions) | problem solving using COPE (creativity, optimism, planning, expert information) | hospital and private oncology center and via telephone | Researcher (no further details) | None identified | No. The intervention has been trialled in other countries, however. |
| Belgacem et al., 2013 | Individual education session: (1) information provision; (2) task demonstrations by nurse; (3) caregiver performs tasks with nurse guidance; and (4) caregiver performs task without supervision, followed with nurse debriefing. | meal support, nursing care (wash and comfort care, and safety care), welfare care (massage), and symptom management | hospital ward | Nurses | None identified | No. The opinions of caregivers, patients, and healthcare providers about an educational programme were surveyed, however. |
| Bultz et al., 2000 | 6 group sessions | information provision about medical and psychosocial aspects of condition, and group therapy | breast cancer clinic | Experienced psychologists | None identified | Not reported |
| Carter, 2006 | 2 individual sessions | Caregiver Sleep Intervention (CASI) incorporating stimulus control, relaxation therapies, cognitive therapy, and sleep hygiene | convenient place for caregiver | Masters-prepared research nurses | None identified | Project was a feasibility and effectiveness study. |
| DuBenske et al., 2014; Namkoong et al. 2012 | Independent sessions via Internet | Comprehensive Health Enhancement Support System (CHESS) - lung cancer information, communication, and coaching system for caregivers. | caregivers' homes | Staff familiar with using CHESS and the Internet (for training on using CHESS) and experts (within CHESS) | stress and coping theoretical framework | No. The intervention (CHESS) has been extensively studied, however. |
| Fegg et al., 2013 | 6 group sessions | Existential behavioural therapy | university hospital | Behavioural therapists | Unclear | Yes, a pilot was undertaken to evaluate and adapt the therapy manual. |
| Hendrix et al., 2013 | 1 or 2 individual bedside sessions and "how to" book | prevention of infection, maintenance of nutrition and adequate elimination, pain control, caregiver-identified specific care issues | university hospital | Nurses | self-efficacy (Bandura) | Yes, pilot studies have been published. |
| Hendrix et al., 2016 | 1 or 2 individual bedside sessions and information handouts | knowledge and skills for managing patient symptoms (prevention of infection, management of fatigue, pain control, and maintenance of nutrition and proper elimination), strategies for managing caregiver psychological distress (deep breathing, progressive muscle relaxation, and pleasant imagery) | university hospital | Nurses | self-efficacy (Bandura) | Partially. The component on knowledge and skills for managing patient symptoms was piloted (see Hendrix et al., 2013), but not the component on strategies for managing caregiver psychological distress. |
| Holm et al., 2016; Holm et al., 2017 | 3 group sessions | promotion of preparedness for caregiving through knowing, being, and doing (topics: palliative diagnoses and symptom relief, daily care and nutrition problems, and support and existential issues) | specialized palliative home care settings | Health professionals (physician, nurse, and social worker/priest); nurses acted as group leaders in all meetings | relatives involvement in palliative care (Andershed & Ternestedt, 2001) | Not reported |
| Hudson et al., 2005 | 2 home visits plus a follow-up phone call between visits, guidebook, and audiotape | education on common caregiving issues, opportunities to access information on relevant issues and skill development, caregiver support (palliative care service, other services, family and friends), normalising emotional reactions, promotion of self-care, provision of options (identifying issues, planning goals/strategies, advising caregivers of rights) | caregivers' homes and via telephone | Nurses | transactional model of stress and coping (Lazarus & Folkman) | Yes, a pilot phase was conducted, but not reported. |
| Hudson et al., 2013; Hudson et al., 2015 | 1 home visits and 3 phone calls (Intervention 1) or 2 home visits and 2 phone calls (Intervention 2) | strategies targeting feelings of preparedness, competence, having adequate information, fewer unmet needs and focusing on positive aspects of the role | caregivers' homes and via telephone | Nurses | transactional model of stress and coping (Lazarus & Folkman) | Yes, Hudson et al., 2005 (included study) referred to as the pilot. |
| Kurtz et al., 2005; Given et al. 2006 | 5 in-person and 5 telephone contacts | managing patient symptoms, reducing emotional distress | oncology centre and via telephone | Nurses | self-efficacy (Bandura) | Not reported |
| Laudenslager et al., 2015; Simoneau et al., 2017 | 8 individual sessions plus workbook | stress management focused on understanding stress, changing role(s) as caregiver, cognitive behavioural stress management, pacing respiration, and identifying social support, plus biofeedback device | hospital/clinic | Masters-level social worker | transactional model of stress and coping (Lazarus & Folkman) | Yes, two pilot studies were conducted. |
| Lee et al., 2016 | face-to-face sessions and telephone calls on alternate weeks | coping strategies, assistance, recourses, education | hospital and via telephone | Cancer nurse with 12 years experience | self-efficacy (Bandura) | Yes, pilot study mentioned as the source of information used to calculate power. |
| Leow et al., 2015 | 1 one-hour face-to-face session (including video clip and development of a care plan), 2 follow-up phone calls, an invitation to an online social support group | coping with stress, frustration, depression, and anticipatory grief; communication between patient and caregiver; social support for caregiver; information on advanced care planning and community resources | caregivers' homes and via telephone | Nurse researcher | self-efficacy (Bandura) | Study was a pilot. |
| Manne et al., 2004 | 6 structured group sessions | health topics (information about prostate cancer treatments, potential sexual side effects of treatments, and healthy nutrition), psychological topics (based on stress and coping theory [Lazarus, 1984] and cognitive and social processing theories of adaptation to difficult life events [Horowitz, 1986; Janoff-Bulman, 1989; Pennebaker, 1989]) | outpatient clinic | Radiation oncologist, nutritionist, psychologist, social worker | stress and coping theory [Lazarus, 1984] and cognitive and social processing theories of adaptation to difficult life events [Horowitz, 1986; Janoff-Bulman, 1989; Pennebaker, 1989] | Not reported |
| Mahendran et al., 2017 | 4 weekly group sessions | psychoeducation, skills training, supportive therapy | outpatient clinics | Clinical psychologist | principles of Brief Integrative Psychological Therapy | Study was a pilot. |
| McMillan et al., 2006; McMillan et al., 2007 | 1 individual session | problem solving using COPE (creativity, optimism, planning, expert information) | caregivers' homes | Nurses | None identified | Not reported |
| Mitchell et al. 2013 | 2 checklist-guided GP consultations | a carer-reported needs checklist and a supporting GP Toolkit of resources (informational, physical, psychological, spiritual, existential, social, financial and legal domains) | GP practices | GPs | None identified | Partially. The checklist was piloted with practicing GPs and actor-patients. |
| Pailler et al. 2015 | 1 group session with handouts, a guidebook, a list of resources, a calendar/planner, and a relaxation CD | medical information, caregiver coping, resources, plus opportunities for networking and support | hospital | Not reported | None identified | Not reported |
| Rexilius et al., 2002 | (a) six 30min massage therapy treatments or (b) six 30min Healing Touch treatments | (a) massage therapy or (b) Healing Touch treatment | oncology outpatient clinic | Certified massage therapist, certified Healing Touch practitioner | None identified | Not reported |
| Shaw et al., 2016 | 4 telephone calls | manualized, standardized assessment of caregiver need (domains: patient care, maintaining family relationships, and emotional and physical self-care) and an assessment of information and practical needs, with information about resources and strategies that might address identified needs | via telephone | clinical psychologists with training in clinical aspects of cancer care | None identified | No. Pilot work was undertaken on identifying caregivers unmet needs and how they perceive their role. |
| Sun et al. 2015 | assessment, interdisciplinary care meeting review, 4 education sessions | (1) quality of life assessments with caregivers and patients were conducted, with results transferred to a personalised palliative care plan; (2) cases (including plans) were presented at weekly interdisciplinary care meetings; and (3) educational sessions focused on physical, psychological, social, and spiritual domains | comprehensive cancer center | Interdisciplinary (nurses, palliative medicine clinicians, thoracic surgeons, medical oncologists, a geriatric oncologist, a pulmonologist, a social worker, a chaplain, a dietitian, a physical therapist) | None identified | Not reported |
| Toseland et al., 1995; Blanchard et al., 1996 | 6 individual counselling sessions | support, problem solving, coping skills | oncology department of a regional medical centre | Experienced oncology social worker | None identified | Not reported |
| Tsianakas et al., 2015 | 1 group consultation with DVD and booklet | treatment side effects, impact of being a caregiver, and dealing with emotions and importance for caregivers of taking time out for themselves and accessing support | hospital | Nurses | None identified | Not reported |
| Walsh et al., 2007 | 6 visits over 6 weeks | advice, information, and emotional support provision with respect to domains of caregiver need (patient care; physical health needs; need for time away from the patient in the short term and longer term; need to plan for the future; psychological health, relationships and social networks; relationships with health and social service providers; finances) | places convenient for caregivers (including telephone calls) | Caregiver advisors (community nurses/social workers) | None identified | Yes |

Supporting Table 2: The acceptability, adoption, and appropriateness of interventions.

| Study | Acceptability | | |  | Adoption |  | Appropriateness | |
| --- | --- | --- | --- | --- | --- | --- | --- | --- |
|  | Data collected on acceptability from caregiver’s perspective | Data collected on acceptability from other stakeholders | Caregiver input into intervention development |  | Evidence of intention, agreement, or action to try to employ intervention |  | The right intervention for the target group (good fit) | Targeting of high risk caregivers through screening or eligibility criteria |
| Bahrami & Farzi, 2014 [34] | No | No | No |  | No |  | Yes, intervention tailored to population needs | No |
| Belgacem et al., 2013 [35] | No | No | No |  | No, and healthcare providers had reservations about what they had to teach the caregivers. |  | Yes, intervention was tailored | No |
| Bultz et al., 2000 [36] | Yes, an open-ended evaluation (survey) found that 79% of caregivers described the intervention a worthwhile and helpful, with 57% reporting they would have liked more sessions and of longer duration. | Yes, patients responded to an open-ended evaluation (survey), indicating that the intervention assisted their partners to be better caregivers (86%), promoted an increase in communication (57%), and helped to improve their relationships (43%). | No |  | No |  | Yes, intervention tailored to the needs of the group | No |
| Carter, 2006 [37] | Yes, debriefings found that caregivers were willing and able to perform the tasks to improve sleep quality, intervention was easy to follow, intervention was responsive to caregivers' energy levels and time constraints, flexibility of delivery of training was advantageous, individualisation of the intervention goal setting was helpful. | No | No |  | No |  | Yes, intervention was tailored | Yes, those with sleep difficulties for at least one month |
| DuBenske et al., 2014; Namkoong et al. 2012 [38, 39] | No | No, but data may have been in other CHESS studies. | No |  | No |  | Yes, intervention allowed self-directed engagement with CHESS | No |
| Fegg et al., 2013 [40] | No | No | No |  | No |  | Yes, intervention addresses caregiver distress | No |
| Hendrix et al., 2013 [41] | Yes, survey conducted within pilot study, showing that the content was relevant to caregivers, the bedside training enabled the skills to be practised and confidence increased, and the individualised approach and flexibility of scheduling was valued (Hendrix & Ray, 2006). | No | No |  | No |  | Yes, intervention tailored towards individual needs | No |
| Hendrix et al., 2016 [42] | Partially, yes for component on knowledge and skills for managing patient symptoms (see Hendrix et al., 2013), no for component on strategies for managing caregiver psychological distress. | No | No |  | No |  | Yes, intervention tailored towards individual needs | No |
| Holm et al., 2016; Holm et al., 2017 [43, 44] | Yes, focus groups conducted, with content perceived as valuable but not always adjusted to caregivers' own situations (Holm et al., 2015). | Yes, focus groups conducted with nurses, physicians, social workers, and priests revealed that the intervention provided satisfaction and insights into their work, but demanded time and engagement from them (Holm et al., 2015). | No |  | No |  | Yes, intervention tailored to group needs | No |
| Hudson et al., 2005 [45] | No | No | Informed development |  | No |  | Yes, intervention tailored to population needs | No |
| Hudson et al., 2013; Hudson et al., 2015 [46, 47] | No | No | Informed development |  | No |  | Yes, intervention tailored to population needs | No |
| Kurtz et al., 2005; Given et al. 2006 [48, 49] | No | No | No |  | No |  | Yes, intervention tailored to patients' individual symptoms and caregiver success in assisting to manage those symptoms. | No |
| Laudenslager et al., 2015; Simoneau et al., 2017 [50, 51] | Yes, feedback was mixed (i) the majority attended the 8 sessions, but did not use the biofeedback device regularly; (ii) the biofeedback device was unacceptable to many caregivers, perhaps due to its bulkiness or the time involved in its use (15min/session); (iii) some did not like the music accompanying the breathing exercise and had trouble syncing their breathing with commands; and (iv) some did not find the device helpful. | No | Informed development |  | No |  | Yes, intervention tailored to population needs | No |
| Lee et al., 2016 [52] | No | No | No |  | No |  | Yes, intervention could accommodate individual needs | No |
| Leow et al., 2015 [53] | Possibly, 3 former caregivers were invited to validate the Caring for the Caregiver Programme prior to commencement - no further details provided. | Possibly, 1 palliative care doctor and 2 palliative care nurses were invited to validate the Caring for the Caregiver Programme prior to commencement - no further details provided. | Informed development |  | No |  | Yes, intervention designed to be tailored to individual needs | No |
| Manne et al., 2004 [54] | No | No | No |  | No |  | Yes, intervention tailored to population needs | No |
| Mahendran et al., 2017 [55] | Yes, interview feedback was that the groups provided knowledge and were safe spaces for self-expression, experiences were normalized, there was experiential learning of coping skills, and negative cognitions were challenged; caregivers accentuated the importance of the physical venue, and would have liked longer sessions, more contextualized examples, more time for caregivers to share with one another, and greater homogeneity within groups in terms of cancer types, stages, and relationship statuses. | No | Informed development |  | No; authors suggested that providing the intervention at no cost may not be sustainable in the long run; integration into routine service delivery may be a solution. |  | Yes, intervention tailored to population needs | No |
| McMillan et al., 2006; McMillan et al., 2007 [56, 57] | No | No | No |  | No |  | Yes, intervention tailored to population needs | No |
| Mitchell et al. 2013 [58] | Possibly, caregivers were interviewed during the development of the intervention - no further details provided. | Partially, stakeholders were interviewed during the development of the intervention and a reference group of practising GPs reviewed the Toolkit for relevance and utility before being finalised. | Informed development |  | No |  | Yes, intervention tailored towards individual needs | No |
| Pailler et al. 2015 [59] | Yes, survey feedback after intervention was that caregivers rated its helpfulness highly; caregivers reported that the program was well organised with appropriate information (especially medical/treatment information), and appreciated the informal, small-group format, which provided the opportunity to ask questions, and program materials were identified as a helpful reference. | No | No |  | No |  | Yes, intervention designed to address unmet needs | No |
| Rexilius et al., 2002 [60] | Yes, survey. Massage: most caregivers indicated the therapy provided time out and relaxation, and some indicated that scheduling massages was difficult, because they felt obligated to stay with the patients. Healing Touch: most reported that the treatments were relaxing. | No | No |  | No |  | Yes, intervention tailored to population needs | No |
| Shaw et al., 2016 [61] | Yes, interviews confirmed the intervention was acceptable, the timing of the phone calls was appropriate, and the format (telephone contact) was attractive; and caregivers identified specific areas of need that the intervention addressed. | No | Informed development |  | No |  | Yes, intervention designed to be tailored to individual needs | No |
| Sun et al. 2015 [62] | No | No | No |  | No |  | Yes, intervention designed to be tailored to individual needs | No |
| Toseland et al., 1995; Blanchard et al., 1996 [63, 64] | No | No | No |  | No |  | Yes, intervention designed to assist caregivers with their most pressing problems | No |
| Tsianakas et al., 2015 [65] | Yes, focus group findings were that caregivers most valued the information in the DVD, reported that the intervention enhanced their knowledge, and indicated that the intervention legitimised the caregiver role. | Yes, focus group findings were that nurses agreed with caregivers that group dynamics could be compromised if there were too few caregivers, but expressed difficulties with group dynamics; also, materials needed to have included people from diverse ethnic backgrounds. | Yes |  | No |  | Yes, intervention tailored to population needs | No |
| Walsh et al., 2007 [66] | Yes, findings from a survey were that the most valued aspect of the advisor visits was the additional emotional support; some thought that more sessions with the advisor would have been beneficial; and some considered that the intervention came too late in the patient's illness. | No, but specialist palliative care teams were involved in the planning, piloting, and conducing of this work. | Informed development |  | No |  | Yes, intervention designed to be tailored to individual needs | Yes, those scoring high for distress |

Supporting Table 3: The feasibility of interventions.

| Study | Participation of Caregivers Screened | | | |  | Participation of Caregivers in Intervention Condition | | | |
| --- | --- | --- | --- | --- | --- | --- | --- | --- | --- |
|  | People Screened | Eligible (% of those screened meeting selection criteria) | Consented (% of those screened) | Commenced study (% of those screened) |  | Withdrawal rate from intervention (% withdrawal) | Unable to complete intervention (e.g., due to death) (%) | Completers (%) | Participant time commitment required for full intervention delivery |
| Bahrami & Farzi, 2014 | Not reported | Not calculable | Not calculable | Not calculable |  | Not reported | Not reported | Not reported | 150mins |
| Belgacem et al., 2013 | 265 patients | 33% | 25% | 25% |  | 0% | 0% | 100% | Unclear (education lasted as long as the caregiver needs to perform care independently) |
| Bultz et al., 2000 | 118 caregiver-patient dyads | 100% | 31% | 31% |  | 0% | 0% | 100% | 12 hrs (6 weekly meetings) |
| Carter, 2006 | 35 caregivers recruited from community settings | 86% | 86% | 86% |  | 0% | 0% | 100% | 1 hr session + 1 hr booster session |
| DuBenske et al., 2014; Namkoong et al. 2012 | 518 caregiver-patient dyads | 100% | 70% | 62% |  | 18% | 23% | 59% | Using CHESS once per week was encouraged, but no mandatory use was required; 59% of participants opted to receive training on using the Internet or Chess |
| Fegg et al., 2013 | 1324 relatives | 89% | 12% | 12% |  | 14% | 0% | 86% | 22 hrs (6 meetings) |
| Hendrix et al., 2013 | 763 patients | 65% | 16% | 16% |  | 0% | 0% | 100% | 2-3 hrs |
| Hendrix et al., 2016 | 3,088 patient records | 22% | 6% | 4% |  | 0% | 0% | 100% | 1-2 hrs |
| Holm et al., 2016; Holm et al., 2017 | 70-200 patients in each of 10 settings | Not calculable | Not calculable | Not calculable |  | 0% | 0% | 100% | 6 hrs (3 weekly 2 hr sessions) |
| Hudson et al., 2005 | 684 caregivers | 52% | 15% | Not calculable |  | Not reported | Not reported | Not reported | Not reported |
| Hudson et al., 2013; Hudson et al., 2015 | 1,163 caregivers (an additional 520 were approached but did not respond) | 64% | 26% | Not calculable |  | Not reported | Not reported | Not reported | Not reported |
| Kurtz et al., 2005; Given et al. 2006 | 609 patients | 90% | 43% | 39% |  | Not reported | Not reported | Not reported | Not reported |
| Laudenslager et al., 2015; Simoneau et al., 2017 | 267 caregivers | 84% | 56% | 55% |  | 7% | 3% | 91% | 10 hrs (eight 60-75min sessions) |
| Lee et al., 2016 | Not reported | Not calculable | Not calculable | Not calculable |  | 0% | 0% | 100% | 15-20min for initial session, 30-40min sessions every 2 weeks, telephone call on alternate weeks per caregiver (sessions continued until patient death) |
| Leow et al., 2015 | 86 caregivers | Not calculable | 93% | Not calculable |  | Not reported | Not reported | Not reported | 90-120mins |
| Manne et al., 2004 | 120 spouses/partners | 100% | 57% | Not calculable |  | Unclear | Unclear | Unclear | 6 hrs (6 weekly 1 hr sessions) |
| Mahendran et al., 2017 | Not reported | Not calculable | Not calculable | Not calculable |  | 21% | 0% | 79% | 4 hrs (4 weekly 1 hr sessions) |
| McMillan et al., 2006; McMillan et al., 2007 | Not reported | Not calculable | Not calculable | Not calculable |  | 0% | 0% | 100% | 1 hr 45 mins (visit 1 = 45 mins; visits 2–3 = 30 mins each) |
| Mitchell et al. 2013 | 872 carers | 88% | 45% | 38% |  | Unclear | Unclear | Unclear | 2 long GP consultations, plus time to complete checklist |
| Pailler et al. 2015 | 180 patients | 74% | 51% | 51% |  | 13% | 0% | 87% | 90min |
| Rexilius et al., 2002 | Not reported | Not calculable | Not calculable | Not calculable |  | 21% | 0% | 79% | 3hrs |
| Shaw et al., 2016 | 385 patients | Not calculable | Not calculable | Not calculable |  | 6% | 6% | 88% | <~2hrs |
| Sun et al. 2015 | Not reported | Not calculable | Not calculable | Not calculable |  | Not reported | Not reported | Not reported | ~112mins plus assessment time |
| Toseland et al., 1995; Blanchard et al., 1996 | 346 caregiver-patient dyads | 93% | 25% | 25% |  | 9% | 23% | 68% | 6 hrs |
| Tsianakas et al., 2015 | 269 patients | 30% | 17% | 17% |  | 8% | 0% | 92% | 79mins |
| Walsh et al., 2007 | 1577 patients | 23% | 17% | 17% |  | Not reported | Not reported | Not reported | Not reported |

Supporting Table 4: The fidelity and costs of interventions.

| Study | Fidelity | | |  | Costs | | |
| --- | --- | --- | --- | --- | --- | --- | --- |
|  | Whether the intervention ran as intended | Dose delivered (% of those completing all intervention sessions) | Changes to the intervention during the study |  | Staff time commitment required for delivery | Additional resources used | Staff training and expertise required to deliver intervention |
| Bahrami & Farzi, 2014 | Not reported | 100% | None reported |  | 150mins | None reported | Not specified |
| Belgacem et al., 2013 | Yes | 100% | None reported |  | Not reported | None reported | Nursing expertise |
| Bultz et al., 2000 | Yes | 80% | None reported |  | 12 hrs | None reported | Counselling psychology skills |
| Carter, 2006 | Yes, first session of each group for each research nurse was observed for fidelity | 100% | None reported |  | 2 hrs | None reported | Nursing expertise |
| DuBenske et al., 2014; Namkoong et al. 2012 | Yes | Not calculable (no denominator) | None reported |  | Not reported | Participants in intervention condition received a laptop computer and Internet access if required | Familiarity with CHESS, the Internet, and operating laptops |
| Fegg et al., 2013 | Yes, therapist adherence to the manual was high | Not reported | None reported |  | 22 hrs | None reported | Behavioural therapists received 20 hrs training in EBT plus supervision |
| Hendrix et al., 2013 | Yes, fidelity of the intervention was monitored | 100% | None reported |  | 2-3 hrs per caregiver | Caregiver written material | Nursing expertise |
| Hendrix et al., 2016 | Yes, fidelity of the intervention was monitored | 100% | None reported |  | 1-2 hrs per caregiver | Caregiver written material | Nursing expertise |
| Holm et al., 2016; Holm et al., 2017 | Yes (intervention was manualised, but no report on fidelity) | 100% | None reported |  | 6 hrs per group | None reported | Expertise of professions involved (physician, nurse, and social worker/priest) |
| Hudson et al., 2005 | Not reported | Not reported | None reported |  | Not reported | Caregiver written and audio material | Nursing expertise |
| Hudson et al., 2013; Hudson et al., 2015 | Not reported | Not reported | None reported |  | Not reported | None reported | Nursing expertise |
| Kurtz et al., 2005; Given et al. 2006 | Yes, quality assurance was performed for all nurses | Not reported | None reported |  | Not reported | None reported | Nursing expertise |
| Laudenslager et al., 2015; Simoneau et al., 2017 | Yes, interventionist fidelity to the manual (>90%) was randomly checked by video for 20% of sessions | 70% | None reported |  | 12-14 hrs | Written material (workbook), biofeedback device | Masters-level social worker |
| Lee et al., 2016 | Not reported | Not reported | None reported |  | 15-20min for initial session, 30-40min sessions every 2 weeks, telephone call on alternate weeks per caregiver (sessions continued until patient death) | None reported | Nursing expertise |
| Leow et al., 2015 | No, participants did not engage with the online forum | 32% | None reported |  | 75-90mins | DVD | Nursing |
| Manne et al., 2004 | Yes, average adherence was >80% | Not reported | None reported |  | 6 hrs | None reported | Radiation oncology, nutrition, psychology, social work expertise |
| Mahendran et al., 2017 | Yes, participants completed the intervention | 100% | None reported |  | 4 hrs | None reported | Clinical psychology expertise |
| McMillan et al., 2006; McMillan et al., 2007 | Yes, intervention was manualised and audio recordings of sessions were reviewed monthly | 100% | None reported |  | Not reported | Home help aide provided respite so caregivers could focus on the intervention | Nursing expertise |
| Mitchell et al. 2013 | Not reported | Not reported | None reported |  | 2 long consultations per caregiver | Written material | general practitioner |
| Pailler et al. 2015 | Not reported | 87% | None reported |  | 90min | Written material (handouts, guidebook, list of resources, and calendar/planner) and audio material (relaxation CD) | Not specified |
| Rexilius et al., 2002 | Not reported | Not reported | None reported |  | 3hrs | None reported | Massage and Healing Touch expertise |
| Shaw et al., 2016 | Yes, mean call length ranged from 32min at 2 weeks to 17min at week 10l; high levels of participation in the intervention calls among caregivers; participants were able to reschedule calls to maximize the number of participants receiving four calls within the 10-week intervention | Not reported | None reported |  | <~2hrs | None reported | clinical psychologists with training in clinical aspects of cancer care |
| Sun et al. 2015 | Not reported | Not reported | None reported |  | ~132mins per caregiver (~20mins for interdisciplinary review, ~28mins for education session) plus assessment time | Written material (manual with teaching content) | Interdisciplinary |
| Toseland et al., 1995; Blanchard et al., 1996 | Yes, sessions were audio-recorded and 10% of sessions were checked for treatment integrity | Not reported, mean number of sessions attended was 5.5 of 6 | None reported |  | 6 hrs | None reported | Counselling skills and experience in oncology |
| Tsianakas et al., 2015 | Not reported | 92% | None reported |  | 79mins per ≤5 caregivers | Written material (booklet), DVD | Nurses trained in group facilitation |
| Walsh et al., 2007 | Yes, advisors met weekly with the research team for debriefing, advice, and to ensure that all domains of caregiver need were being covered | 83% | None reported |  | Not reported | None reported | Community nursing and/or social work |
